# Comparative biomechanical analysis of tibial posterior slope in medial open wedge high tibial osteotomy vs. distal tuberosity osteotomy with and without anterior-posterior screw: a study using porcine tibia

**DOI:** 10.1051/sicotj/2024042

**Published:** 2024-10-21

**Authors:** Yoshiya Nibe, Tsuneari Takahashi, Hironari Hai, Tomohiro Matsumura, Katsushi Takeshita

**Affiliations:** 1 Department of Orthopaedics Surgery, Jichi Medical University 3311-1 Yakushiji Shimotsuke 329-0498 Japan; 2 Department of Orthopaedic Surgery, Toyokawa City Hospital 23 Noji Toyokawa 442-0857 Japan; 3 Department of Emergency and Critical Care Medicine, Jichi Medical University 3311-1 Yakushiji Shimotsuke 329-0498 Japan

**Keywords:** Biomechanical study, High tibial osteotomy, Distal tuberosity osteotomy, Porcine tibia, Posterior tibial slope

## Abstract

*Purpose* While increased posterior tibial slope (PTS) is a concern post-medial open wedge high tibial osteotomy (MOWHTO), the ability of distal tuberosity osteotomy (DTO) to maintain postoperative PTS after cyclic loading remains unverified. This study aims to determine whether PTS alterations significantly differ between DTO and MOWHTO following cyclic loading. *Methods*: Biomechanical evaluations were conducted on thirty porcine tibias using MOWHTO and DTO, with and without an anterior-posterior (AP) screw. To investigate PTS changes, cyclic testing was carried out for MOWHTO and DTO. Displacement along the mechanical axis during cycles 10th, 100th, 500th, 1000th, 1500th and 2000th, variations in anterior and posterior gaps after 2000 cycles and increased PTS after 2000 cycles, were compared across the three groups. The displacement was evaluated by repeated-measures analysis of variance (ANOVA), and changes in AG and PG and increased PTS were evaluated by one-way ANOVA. The sample size for α and β errors were <0.05 and <0.20, and the effect size was 0.60 for one-way ANOVA and 0.46 for repeated-measures ANOVA. *Results*: There were no significant differences in displacement and anterior gap changes among the groups. A significant difference was observed in the posterior gap changes (*P* < 0.001) and increased PTS (*P* = 0.013) among the groups. Post hoc analysis indicated substantial disparities between MOWHTO and DTO without the AP screw (*P* = 0.035), as well as between MOWHTO and DTO with the AP screw (*P* = 0.021) concerning the increased PTS. Conclusion: After cyclic loading, MOWHTO exhibited a notably smaller PTS change than DTO regardless of the presence of an AP screw.

## Introduction

Medial open wedge high tibial osteotomy (MOWHTO) is a common treatment for medial knee osteoarthritis [1]. Unfortunately, this treatment is associated with several complications, including postoperative patella baja [2, 3], potentially resulting in the progression of patellofemoral (PF) joint osteoarthritis. Gaasbeek et al. introduced an open wedge distal tuberosity osteotomy known as distal tuberosity osteotomy (DTO) to address this issue [4]. In the MOWHTO method, the tibial tuberosity remains on the proximal fragment. In contrast, the DTO method retains it on the distal fragment. This allows the osteotomy to remain open while preserving patellar height. Since Gaasbeek et al.’s 2004 report on DTO, several studies have found that DTO preserves patellar height [5, 6].

Another complication of MOWHTO involves an increased posterior tibial slope [7, 8]. However, no studies have tried to determine if DTO influences the posterior tibial slope (PTS) after cyclic loading. Therefore, we aimed to investigate whether MOWHTO, DTO without an anteroposterior (AP) screw and DTO with AP screw would provide maintenance of anterior gaps (AG), posterior gaps (PG), and PTS. We hypothesized that MOWHTO would result in less PTS increase than DTO and that a significant difference in PTS increase would be observed between DTO with and without an AP screw.

## Materials and methods

### Study design

**Figure 1:**
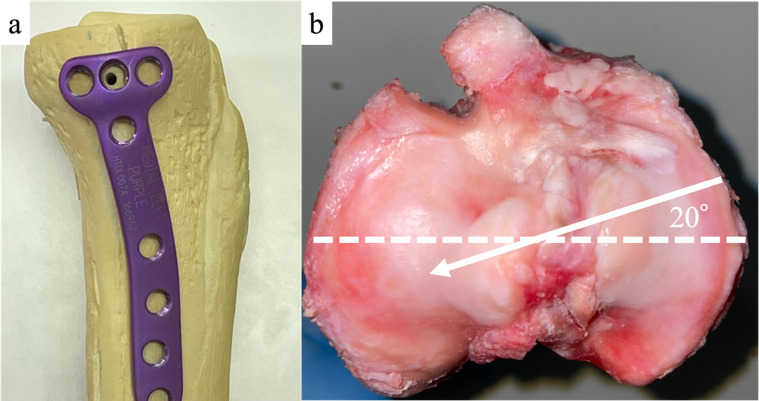
(a) TriS plate (Olympus Terumo Biomaterials, Tokyo, Japan). (b) Anteromedial plate positioning (left knee), with the screw positioned (white arrow) 20° from the transverse diameter of the tibial plateau (dotted line).

All animal experiments were conducted in our institution’s biomechanics laboratory following all Animal Care and Use Committee regulations, although the requirement for ethical approval was waived due to the study’s *ex vivo* design. Thirty fresh porcine knees (age: 6 months; weight range: 180–200 kg; Tokyo Shibaura Zouki, Japan) were divided into three groups. The first group was MOWHTO (Group H) (*n* = 10); the second was DTO without an anteroposterior (AP) screw (Group D1) (*n* = 10); and the third was DTO with an AP screw (Group D2) (*n* = 10). All specimens were fitted with a TriS plate (Olympus Terumo Biomaterial, Tokyo, Japan) (Figure 1a) that was fixed to the medial tibia with the thread direction of the tibial diaphysis set at 20° (anteromedial position) from the transverse diameter of the tibial plateau (Figure 1b), as per a previous study by Takeuchi et al. [9]. The specimens were thawed at room temperature for at least 24 h before use. The medial proximal tibial angle (MTPA) of specimens before MOWHTO and DTO was not excessively valgus or varus macroscopically.

### MOWHTO and DTO procedures

**Figure 2:**
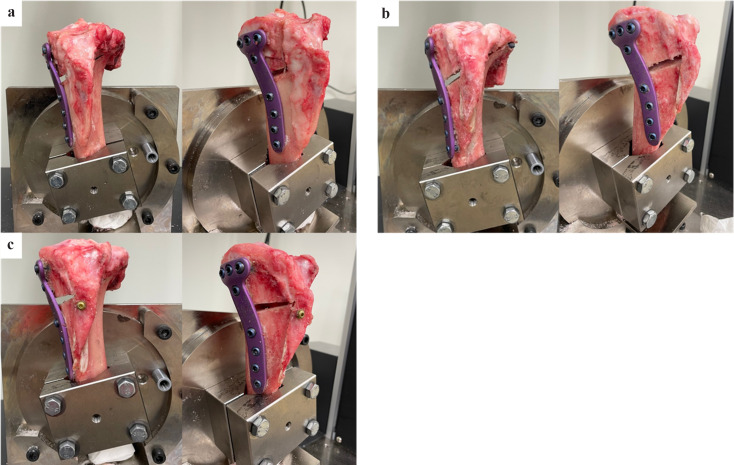
Specimen preparation: (a) specimen after MOWHTO to the left knee; (b) specimen after DTO without AP screw to the left knee; (c) specimen after DTO with AP screw to the left knee. AP: anteroposterior; DTO: distal tuberosity osteotomy; MOWHTO: medial open wedge high tibial osteotomy.

MOWHTO was performed on Group H specimens using standard surgical methods [10] (Figure 2a). First, we inserted a 1.6-mm K-wire 45 mm from the medial joint line and directed it towards the tip of the fibula. We confirmed the K-wire’s direction by puncturing the opposite cortex and touching the fibula’s tip. Further, an oscillating saw was used to perform the osteotomy, following the path of the K-wire from the posterior to the anterior medial tibial cortex. The osteotomy ended 5 mm from the lateral tibial cortex, leaving a hinge for opening. An additional osteotomy of the anterior third of the tibial cortex, including the tibial tuberosity fragment, was executed in the anterior forehead plane at an angle of 110° to the axial plane. We opened the osteotomy with three chisels while applying a bulging gas force to the distal end. A 6-mm gap was identified, and the osteotomy line was maintained parallel. A TriS plate was positioned medially on the proximal tibia and then fixed. Four locking monocortical screws and four bicortical screws were used to secure the proximal and distal bone fragments, respectively. We used a sleeve to guide the screws and ensure they were placed in the same direction. Group 1 had no artificial bone inserted into the open wedge.

DTO was performed on D1 and D2 specimens according to Ogawa et al.’s method [11] (Figures 2b and 2c). We initially conducted a distal tibial tuberosity osteotomy with a thickness of 7–8 mm at the thickest point of the tibial tuberosity [12]. The length of the osteotomised tibial tuberosity was 50 mm. The proximal part of the tibial tuberosity was attached to the proximal fragment of the tibia. Before the transverse osteotomy, a 1.6-mm K-wire was placed 50 mm from the medial joint line and directed towards the tip of the fibula. We then performed a transverse osteotomy at a right angle to the descending osteotomy in the sagittal plane, starting from the tibia’s posteromedial corner (35 mm below the joint line to 7.5 mm from the lateral cortical margin at the level of the fibula head). We were careful to not disrupt the continuity of the tibial tuberosity and the proximal fragment of the tibia. This transverse osteotomy followed the direction of the K-wire from the posterior to the anterior medial tibial cortex. We carefully opened the osteotomy using three chisels while applying a bulging gas force to the distal end. A 6-mm gap was identified, and the osteotomy line was kept parallel. A TriS plate was placed medially on the proximal tibia and then fixed. Four locking monocortical screws and four bicortical screws were used to secure the proximal and distal bone fragments, respectively. We used a sleeve to guide the screws and ensure they were placed in the same direction. No artificial bone was inserted into the open wedge in Groups D1 and D2. Finally, the tibial tuberosity was fixed to the distal fragment of the tibia with an AP screw in the case of Group D2. Group D1 specimens were not fitted with AP screws.

### Biomechanical evaluation

**Figure 3:**
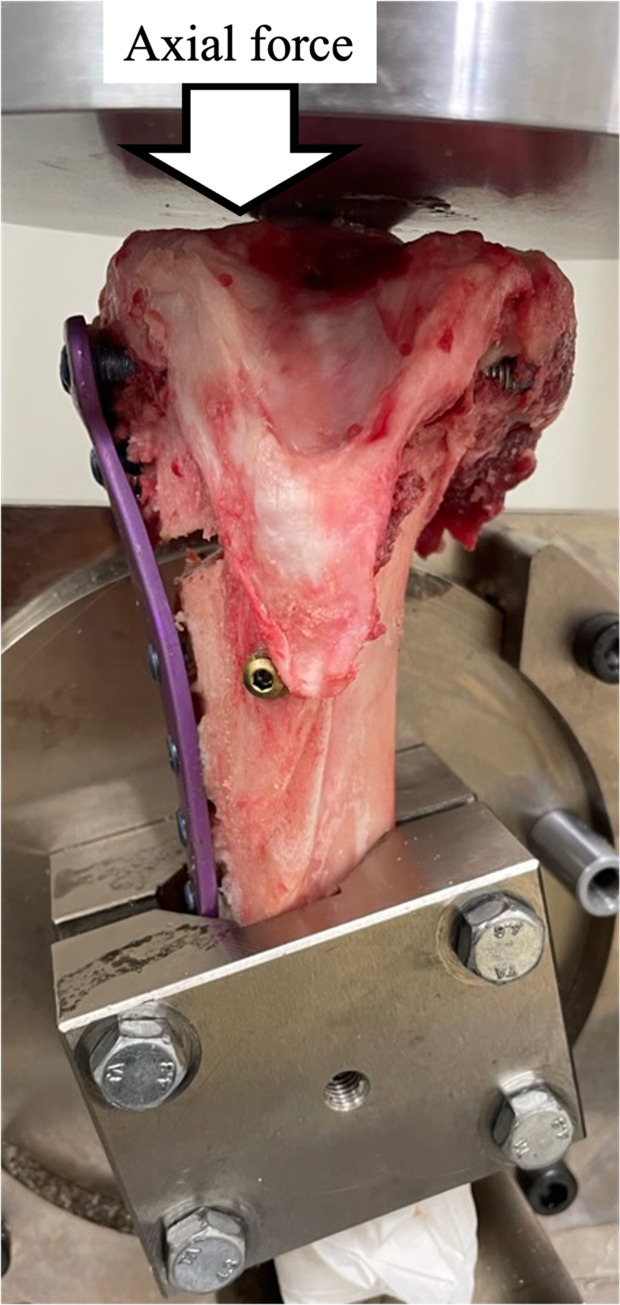
A tibia after DTO with AP screw was clamped with custom-made jig of a tensile testing machine.

**Figure 4:**
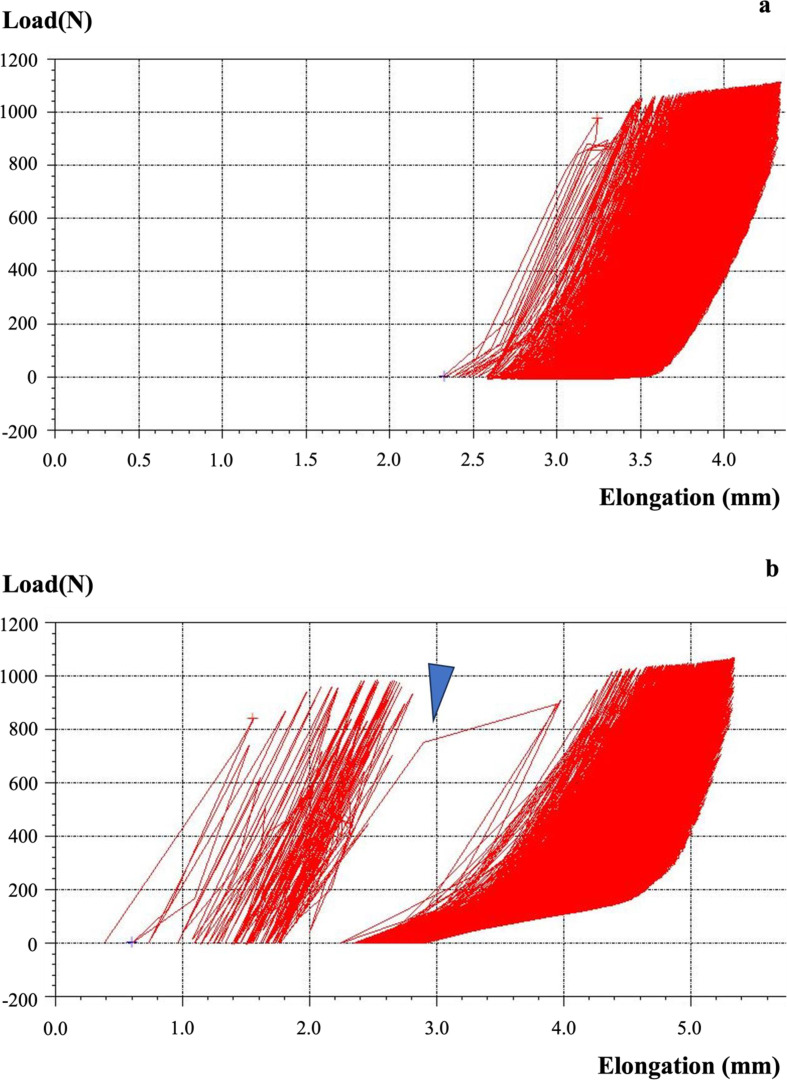
Schematic image of the load-displacement curve generated during cyclic testing with this software. (a) The load-displacement curve of MOWHTO generated during cyclic testing with this software. (b) The load-displacement curve of DTO with AP screw generated during cyclic testing with this software, including a hinge fracture (triangle). AP: anteroposterior; DTO: distal tuberosity osteotomy; MOWHTO: medial open wedge high tibial osteotomy.

We used cyclic testing to investigate the translation patterns of the three fixation constructs. This testing was performed along the postoperative mechanical axis of the tibia using a tensile testing machine (Tensilon RTG 1310, Orientec Co. Ltd., Tokyo, Japan) equipped with a specially designed set of grips. This measurement system aligned with the one used in a prior biomechanical study [13–15]. After removing 10 cm of the distal tibia, 4 cm of the distal portion of each tibia was clamped using a custom-made jig (Figure 3). MTPA after MOWHTO and DTO was about 90°, and the proximal tibial articular after setting was level with the lane of loading macroscopically. Load-displacement curves were generated with specific software (Tensilon Advanced Controller for Testing, Orientec Co., Japan). The tibia was then subjected to cyclic loading up to 800 N (2000 cycles, 0.5 Hz) as previously described by Takeuchi et al. [9]. The displacements in the machine’s axial direction at the 10th, 100th, 500th, 1000th, 1500th, and 2000th cycles were calculated by the software, measuring actuator displacement during the cycle (Figures 4a and 4b). The alignment of specimens after cyclic loading was not excessively altered macroscopically. The AG and PG changes in the osteotomy site were measured using a precision calliper with an accuracy of 0.1 mm. Measurements of the AG and PG changes were taken by two researchers and averaged over three measurements. Therefore, intra- and inter-rater reliability was improved. Increased PTS was calculated by subtracting AG from PG and using the anteroposterior diameter of the proximal tibial articular surface (average 50 mm). The AP diameter of the porcine’s proximal tibial articular was measured at an average of 50 mm; therefore, the AP diameter was calculated at 50 mm in this study.

### Statistical analyses

Repeated-measures analysis of variance (ANOVA) with Bonferroni post hoc analysis was employed to assess displacement among the three groups. A one-way ANOVA with Tukey post hoc analysis was used to evaluate AG and PG changes, and increased PTS among the three groups. All data are presented as mean ± standard deviations. *P*-values <0.05 were considered statistically significant. An *a priori* power analysis was conducted using G*Power 3.1 (Franz Paul, Kiel, Germany) [16]. The sample size for α error was set at <0.05, for β error at <0.20, and the effect size was 0.46 for repeated-measures ANOVA and 0.60 for one-way ANOVA. All statistical analyses were carried out using the EZR software [17]. The minimum sample size by one-way ANOVA of AG and PG changes and increased PTS was 66, and the minimum sample size by repeated-measures ANOVA of actuator displacement was 291. If a significant difference was found with fewer samples than these, it meant that the difference was quite large. Therefore, the effect sizes for the repeated measures and one-way ANOVA were larger than Cohen’s effect size guidelines, indicating that the differences between each group were highly significant.

## Results

### Displacement during cyclic loading

There were no significant differences in displacement for the 10th, the 100th, the 500th, the 1000th, the 1500th and the 2000th cycle among the three groups, respectively (Table 1).

**Table 1:** The displacement among the three groups.

Parameters	Group H (*n* = 10)	Group D1 (*n* = 10)	Group D2 (*n* = 10)
10th (mm)*	0.92 (0.21)	1.08 (0.38)	1.10 (0.28)
100th (mm)*	0.94 (0.24)	1.08 (0.37)	1.23 (0.43)
500th (mm)*	0.93 (0.27)	1.22 (0.52)	1.28 (0.55)
1000th (mm)*	1.01 (0.41)	1.29 (0.56)	1.33 (0.57)
1500th (mm)*	1.02 (0.44)	1.39 (0.61)	1.36 (0.59)
2000th (mm)*	1.02 (0.47)	1.47 (0.60)	1.35 (0.59)

* Data are expressed as mean (standard deviation).

### Changes in the AG and PG

**Figure 5:**
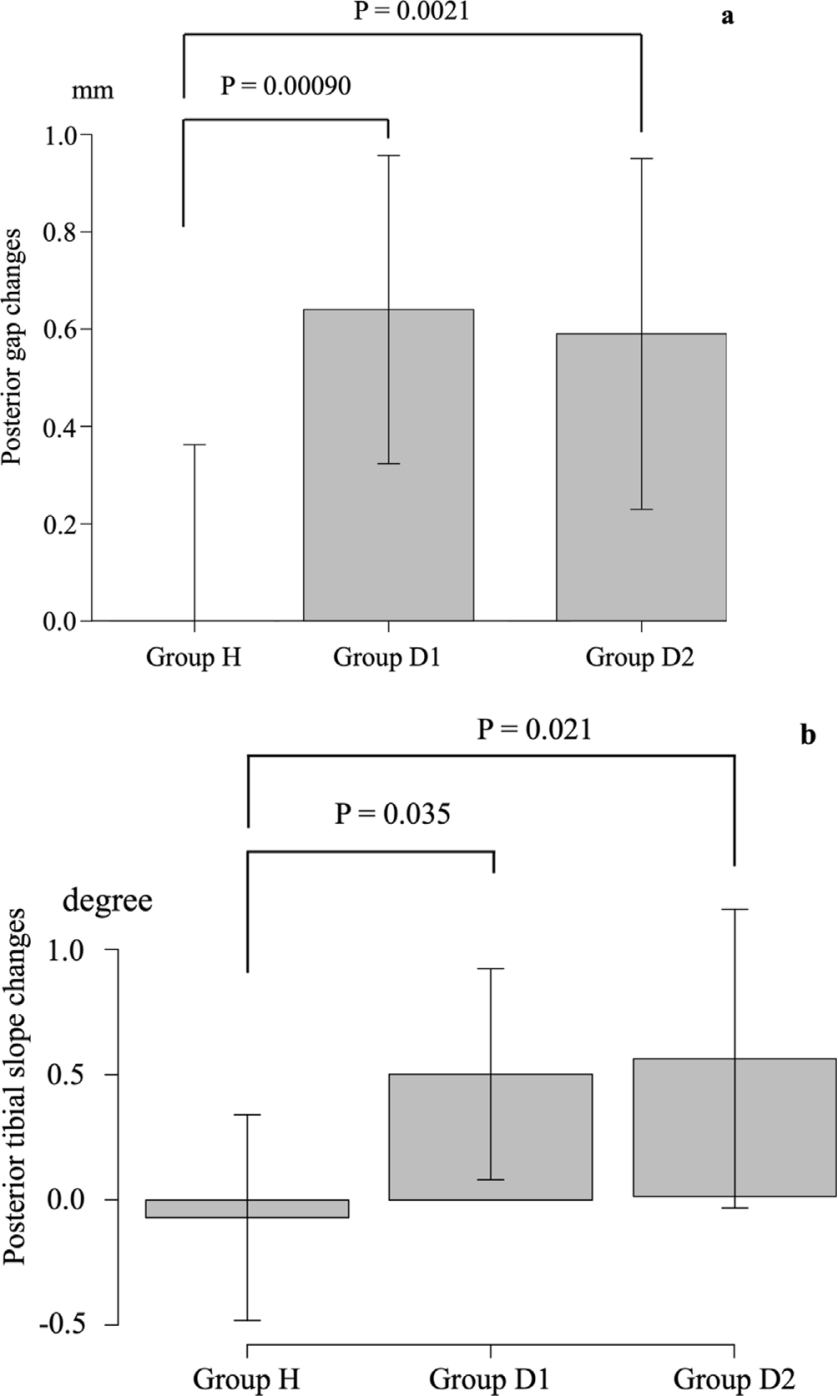
Group-specific displacement: (a) Group-specific changes in the posterior gap, (b) Group-specific changes in the posterior tibial slope.

There were no significant differences in anterior gap changes, respectively (Table 2). However, significant differences were observed in posterior gap changes, respectively (*P* = 0.00042) (Table 2). The post hoc analysis revealed significant differences between Groups H and D1 (*P* = 0.00090) and Groups H and D2 (*P* = 0.0021) (Figure 5a).

**Table 2:** The anterior and posterior gap changes and the posterior tibial slope changes among three groups.

Parameters	Group H (*n* = 10)	Group D1 (*n* = 10)	Group D2 (*n* = 10)	*P* value**
The anterior gap changes*	0.06 (0.32)	0.20 (0.17)	0.11 (0.36)	0.57
The posterior gap changes*	0.00 (0.36)	0.64 (0.32)	0.59 (0.36)	0.00042
The posterior tibial slope changes*	−0.070 (0.41)	0.50 (0.42)	0.55 (0.60)	0.013

* Data are expressed as mean (standard deviation).

** Comparison among Groups by use of one-way analysis of variance.

### Changes in increased PTS

Significant differences were observed in increased PTS, respectively (Table 2). The post hoc analysis revealed significant differences between Groups H and D1 (*P* = 0.035) and Groups H and D2 (*P* = 0.021) (Figure 5b).

## Discussion

In this study, MOWHTO exhibited less of an increase in PTS after cyclic loading than DTO, regardless of the presence or absence of an AP screw. This is the first study to quantitatively compare increases in PTS after cyclic loading following MOWHTO and DTO with and without an AP screw.

We found that the proximal fragment contributed to the increased PTS during cyclic loading after both MOWHTO and DTO. However, the increase in PTS observed following MOWHTO was less pronounced than following DTO regardless of AP screw use. Kim et al. used an anteromedial plate – similar to the TriS plate – and reported an increase in PTS following MOWHTO; however, the increase was less than that observed following DTO in another clinical study [18]. In the previous studies, several investigators generally reported that PTS increased after MOWHTO [19–22], whereas others reported that PTS did not change or decrease after MOWHTO [23, 24] (Table 3).

**Table 3:** List of previous clinical studies about PTS after MOWHTO.

Author	Age	Plate	Result (degrees)	
Yoon et al. [19]	63.6 ± 6.9	Anatomical plate	10.2 ± 2.8	11.2 ± 3.9
Ji et al. [20]	50.4 ± 8.5	TOMOFIX	10.2 ± 3.1	10.5 ± 3.1
Yazdi et al. [21]	42.4 ± 10.0	Puddu plate	16.3 ± 4.2	17.1 ± 4.2
	41.0 ± 9.9	TOMOFIX	16.9 ± 4.7	17.7 ± 5.4
Mabrouk et al. [23]	42.1 ± 12.3	Unknown	9.6 ± 3.4	9.4 ± 3.2
Park et al. [24]	<55	TOMOFIX	10.2 ± 4.4	9.7 ± 4.5
	>65	TOMOFIX	12.1 ± 3.6	11.1 ± 4.1
Li et al. [22]	57.2 ± 7.4	TOMOFIX	8.8 ± 3.7	9.0 ± 3.3

Greater PTS has been linked to strain on the anterior cruciate ligament (ACL) and greater anterior tibial translation in the knees [1, 7]. Furthermore, patients with ACL tears demonstrate increased PTS than knees with normal ACLs [25]. Therefore, increases in PTS may increase the risk of ACL injury. In a biomechanical study of walking movement, an anterior shear force increased by 30% and anterior tibial translation increased by 2.4 mm when PTS increased by 5° [26]. Hence, minimising PTS changes after surgery is crucial. Our results suggest that MOWHTO helps reduce ACL strain after surgery because it produces less of an increase in PTS.

When axial load was applied to the proximal tibial surface in all three groups, there was a noticeable movement of the proximal surface, increasing the PTS. MOWHTO also moves the proximal surface and PTS increases under axial loading. However, the contact between the tibial tuberosity fragment and the anterior surface of the proximal tibia may have mitigated further increases in PTS. Therefore, when an axial load was applied, PTS increased slightly, contacting and pressing against the anterior surface of the proximal tibia and the tibial tuberosity fragment. This contact and compression between the two fragments may have helped accelerate the early bony union.

Nakamura et al. previously reported that MOWHTO with pes anserinus preservation generated natural compression forces between the proximal and tibial tuberous fragments [27]. The authors felt this compression fostered an efficient union involving soft tissues. In this study, soft tissues – including the patellar tendon and pes anserinus – were removed. Despite the absent pes anserinus, which acts as a dynamic stabiliser, MOWHTO still promoted efficient union by transmitting axial loading and the resulting compression forces between the tibial tuberosity fragment and the anterior surface of the proximal tibia.

MOWHTO is an osteotomy designed to reduce the pressure of the medial knee compartment and Habib et al. reported that improvement in femoral and tibial articular cartilage in the medial compartment owing to the unloading effect of MOWHTO and regeneration of fibrocartilage [28]. In contrast, DTO is an osteotomy designed to maintain the patellar height and has been reported to have a lesser impact on the progression of PF osteoarthritis than MOWHTO [5, 6]. Therefore, DTO is a suitable choice in patellar baja or PF osteoarthritis cases [5]. DTO is recommended for young and middle-aged patients with a normal patellar height because it does not alter it [29]. However, Kim et al. reported no significant differences in the occurrence of PF osteoarthritis between MOWHTO and DTO [7, 30]. Double-level osteotomy could be an alternative treatment for DTO because Double-level osteotomy is not a change of patellar height like DTO [31]. Hence, it is debatable which surgical method is better for preventing the progression of PF osteoarthritis. Therefore, a study comparing the clinical outcomes of PF osteoarthritis after MOWHTO and DTO would be valuable.

This study does have several limitations that should be acknowledged. First, results generated from porcine bone experiments may not directly translate into clinical practice as porcine tibias might possess different fixation forces than human tibias. However, it is worth noting that porcine knees are very similar to human knees and, for this reason, are commonly used in biomechanical studies [13, 15, 32]. Second, this study focused solely on time-zero structural characteristics in specimens subjected to MOWHTO or DTO. We did not consider the influence of any biological healing processes. Moreover, the *ex vivo* nature of the study may not fully replicate actual *in vivo* loading conditions. Therefore, future *in vivo* studies are needed to determine if similar results can be achieved in real-life settings. Third, the machine used in this study could only evaluate axial direction. In this study, however, increased PTS can be assessed by measuring the AG and PG. Hence, evaluation in the sagittal direction was also possible. Fourth, there might have been slight malalignment because no radiographic evaluation was performed. Two researchers confirmed that there were no major deformations before and after the cyclic test, and the inter-rater reliability was minimized as much as possible. Finally, the limit of the machine in this study was 2000 cycles. If more cycles had been performed, there might have been differences in displacement and changes in AG.

Despite these limitations, this study represents the first biomechanical comparison of MOWHTO and DTO, both with and without an AP screw, after cyclic loading. From a clinical perspective of this study, MOWHTO is the preferred treatment for internal knee osteoarthritis and ACL insufficiency. Further, in vivo research is needed to delve deeper into this topic.

In conclusion, MOWHTO demonstrated a significantly smaller increase in PTS than DTO, regardless of the presence or absence of AP screws. Considering this result, MOWHTO is the preferred treatment for medial knee osteoarthritis and ACL insufficiency. Therefore, when considering treatment options for knee osteoarthritis in young patients, selecting the appropriate osteotomy method is important, considering other knee joint pathologies.

## Data Availability

Data and materials of this study are available from the corresponding author on reasonable request.
